# Serum nesfatin-1 levels are decreased in pregnant women newly diagnosed with gestational diabetes

**DOI:** 10.1590/2359-3997000000288

**Published:** 2017-09-04

**Authors:** Esra Nur Ademoglu, Suheyla Gorar, Muge Keskin, Ayse Carlioglu, Rifki Ucler, Husamettin Erdamar, Cavit Culha, Yalcin Aral

**Affiliations:** 1 Department of Endocrinology and Metabolism Bolu University Bolu Turkey Department of Endocrinology and Metabolism, Bolu University, Bolu, Turkey; 2 Department of Endocrinology and Metabolism Antalya Education and Research Hospital Antalya Turkey Department of Endocrinology and Metabolism, Antalya Education and Research Hospital, Antalya, Turkey; 3 Department of Endocrinology and Metabolism Ankara Education and Research Hospital Ankara Turkey Department of Endocrinology and Metabolism, Ankara Education and Research Hospital, Ankara, Turkey; 4 Department of Endocrinology and Metabolism Erzurum Education and Research Hospital Erzurum Turkey Department of Endocrinology and Metabolism, Erzurum Education and Research Hospital, Erzurum, Turkey; 5 Department of Endocrinology and Metabolism Van Yuzuncu Yil University Faculty of Medicine Van Turkey Department of Endocrinology and Metabolism, Van Yuzuncu Yil University Faculty of Medicine, Van, Turkey; 6 Department of Biochemistry Turgut Ozal University Faculty of Medicine Ankara Turkey Department of Biochemistry, Turgut Ozal University Faculty of Medicine, Ankara, Turkey

**Keywords:** Gestational diabetes, nesfatin-1, insulin resistance

## Abstract

**Objective:**

To investigate serum nesfatin-1 levels at 24-28 weeks of pregnancy in women newly diagnosed with gestational diabetes and determine the association of nesfatin-1 with several metabolic parameters.

**Subjects and methods:**

Forty women newly diagnosed with gestational diabetes at 24-28 weeks of pregnancy and 30 healthy pregnant women matched in age and gestational week were included in this cross-sectional study. Serum nesfatin-1 levels were analyzed using ELISA, and the relationship between nesfatin-1 and several metabolic parameters were assessed.

**Results:**

Serum nesfatin-1 levels were found to be lower in women with gestational diabetes compared to the pregnant women in the control sample (p = 0.020). Multiple linear regression analysis revealed that nesfatin-1 was lower in participants with gestational diabetes independently from gestational age, BMI, HOMA-IR, fasting plasma glucose, and age. In correlation analysis, the only variable that was found to have a statistically significant correlation with nesfatin-1 was gestational age (p = 0.015, r = 0.30).

**Conclusion:**

Lower nesfatin-1 levels in women with gestational diabetes compared to the control group at 24-28 weeks of gestation draws attention to nesfatin-1 levels in gestational diabetes and motivates further research in this area.

## INTRODUCTION

Gestational diabetes mellitus (GDM) is defined as glucose intolerance of variable degree that begins or is first recognized during pregnancy. Worldwide, the incidence of GDM is gradually increasing every year (1), varying from 3% to 14%. Hyperglycemia during pregnancy is associated with the development of preeclampsia, fetal macrosomia, emergency cesarean section, birth trauma, and neonatal hypoglycemia. GDM is important not only due to its complications but also its conversion rates to Type 2 diabetes mellitus (DM) as high as 2.6% to 70% over a period of 6 weeks to 28 years (2).

Nesfatin-1 is a newly discovered hormone derived from nucleobindin-2 (NUCB2) and has been thought to be involved in the regulation of appetite and various metabolic conditions. It is prominently expressed in several regions of the hypothalamus and exists in the general circulation. Nesfatin-1 is also produced in the peripheral tissues, including adipocytes, gastric mucosa, and pancreatic beta-cells of both humans and rats. One of the most important functions of nesfatin-1 is reducing food intake. It causes loss of appetite, less frequent hunger, and a sense of fullness (3-6). It has been demonstrated that pancreatic beta cells colocalize with nesfatin/NUCB2 in the islets of both mice and rats, indicating the possible involvement of nesfatin-1 in the regulation of insulin secretion from pancreatic beta cells (7). It has been shown that fasting nesfatin-1 levels were significantly lower in type 2 diabetic patients, but its effects on those who have newly been diagnosed with GDM are unknown (8).

In this study, we aimed to examine serum nesfatin-1 concentrations and to investigate whether they have any correlation with insulin resistance, and to examine other metabolic parameters in patients who had newly been diagnosed with gestational diabetes.

## SUBJECTS AND METHODS

Forty women newly diagnosed with GDM at 24-28 weeks of pregnancy and 30 healthy pregnant women matched in terms of age and gestational age was enrolled in the study. Diagnosis of GDM was based on 2015 American Diabetes Association recommendations (9). A two-step approach to screening with a 1-h 50-g glucose load test (GLT) followed by a 3-h 100-g OGTT for those who screened positive was used in examining all individuals. Pregnant women who had glucose levels higher than 7.8 mmol/l one hour after a 50-g glucose-screening test were referred to a 3-hour, 100-g oral glucose tolerance test and Carpenter/Coustan thresholds were used. GDM was diagnosed if two or more of the following plasma glucose levels were exceeded: fasting ≥ 5.1 mmol/l, 1-hour ≥ 10 mmol/l, 2-hour ≥ 8.6 mmol/l, and 3-hour ≥ 7.8 mmol/l (9). Exclusion criteria for the study were known pregestational diabetes, liver or renal dysfunction, acute or chronic inflammatory diseases, pre-eclampsia, use of anti-inflammatory drugs, and smoking. The body mass index was calculated for all participants [BMI: body weight (kg)/squared height (m^2^)]. The ethical committee of Ankara Education and Research Hospital approved the study. A written informed consent was obtained from all the participants.

Blood sampling was conducted in the morning following one night of fasting and centrifuged immediately. Serum samples were stored at −80ºC until the time of analysis. Serum nesfatin-1 concentrations were analyzed with ELISA kits from USCN Life Science Instruments (Wuhan, China). In this assay system, the intra-assay and inter-assay coefficient of variation are always under 10%. This assay employs the competitive inhibition enzyme immunoassay technique. A monoclonal antibody specific for human NES1 was pre-coated onto a microplate. A competitive inhibition reaction was launched between biotin-labeled human NES1 and unlabeled human NES1 (standards or samples) with the pre-coated antibody specific for human NES1. After incubation, the unbound conjugate was washed off. Next, avidin conjugated to horseradish peroxidase (HRP) was added to each microplate well and incubated. The amount of bound HRP conjugate was reverse proportional to the concentration of NES1 in the sample. After addition of the substrate solution, the intensity of color developed was reverse proportional to the concentration of NES1 in the sample. Serum glucose was assayed with a hexokinase method using an Olympus AU 2700 analyzer (Olympus UK, London, UK), and hemoglobin A1c (HbA1c) was measured with high-performance liquid chromatography (HLC-723 G7 HPLC systems; Tosoh Corporation., Tokyo, Japan). Serum insulin was determined using a sandwich enzyme-linked immunosorbent assay (ELISA) (Dynex DSX full automatic ELISA analyzer, USA). Insulin resistance (IR) was calculated for each patient using the homeostasis model assessment insulin resistance index (HOMA-IR), fasting plasma glucose (mmol/l) × fasting insulin (mIU/mL)/22.5.

In all statistical analyses, SPSS software version 15.0 was used. Distributions were tested for normality using the Shapiro-Wilk test. The normally distributed variables were analyzed with the Student’s t-test, and the data variables that did not show a normal distribution were compared with the Mann-Whitney U-test. Bivariate correlations between nesfatin-1 and several parameters were analyzed by Pearson correlation test for normally distributed variables. Spearman correlation test was used for nonnormally distributed variables. Multiple regression analysis was used to exclude the possible confounding effect of other variables on the result of each correlation analysis. P values less than 0.05 were considered statistically significant for all statistical analyses. The data for continuous variables were presented as mean ± standard deviation.

## RESULTS

Demographical characteristics and biochemical values of women with GDM and healthy controls are shown in Table 1. The participants with GDM and the control group were similar in terms of mean age (29.6 ± 5.3 vs. 27.8 ± 6.0 years, respectively). Serum fasting glucose, 1-h glucose after 50-g glucose screening test, 1-h glucose, 2-h glucose, and 3-h glucose after 100-g oral glucose tolerance test, HbA1c, and BMI were higher in women with GDM than those in the control group (p < 0.05). Fasting insulin and HOMA-IR were similar between the two groups. Serum nesfatin-1 concentrations were significantly found to be lower in the participants with GDM compared to the controls (7.9 ± 2.8 vs. 11.2 ± 7.7 ng/mL, respectively, p = 0.020, Table 1).

**Table 1:** Demographical characteristics and biochemical values of controls and women with gestational diabetes

	Controls (n = 30)	Gestational diabetes (n = 40)	p value
Age, year	27.8 ± 6.0	29.6 ± 5.3	0.242
Gestational age (week)	25.9 ± 1.5	26.2 ± 1.8	0.443
BMI (kg/m^2^)	28.2 ± 1.5	31.0 ± 5.5	0.018^a^
Fasting insulin (pmol/l)	55.2 ± 38.0	62.4 ± 38.2	0.441
HOMA-IR	1.4 ± 0.7	2.0 ± 1.7	0.074
50 g OGTT			
Glucose 1 h (mmol/l)	6.7 ± 1.7	10.3 ± 2.2	0.000^a^
100 g OGTT			
Glucose 0 h (mmol/l)	4.0 ± 0.4	4.8 ± 0.8	0.001^a^
Glucose 1 h (mmol/l)	9.1 ± 0.4	11 ± 1.4	0.000^a^
Glucose 2 h (mmol/l)	7.6 ± 1.9	9.4 ± 2.3	0.000^a^
Glucose 3 h (mmol/l)	5.7 ± 0.9	6.2 ± 2.2	0.004^a^
HbA1c (mmol/mol)	32	36	0.004^a^
(%)	4.8 ± 1.0	5.9 ± 1.1	
Nesfatin-1 (ng/mL)	11.2 ± 7.7	7.9 ± 2.8	0.020^a^

BMI: body mass index; HOMA-IR: homeostasis model assessment insulin resistance index.

^a^ The difference between controls and gestational diabetes was statistically significant (p < 0.05).

Multiple linear regression analysis revealed that nesfatin-1 was lower in women with GDM independent of gestational age, BMI, age, fasting plasma glucose, and HOMA-IR (Table 2). Nesfatin-1 levels had a statistically significant positive correlation with gestational age (r = 0.30, p = 0.015, Table 3). There was not any correlation between nesfatin-1 and BMI, HOMA-IR, fasting glucose, 1h-glucose after 50-g glucose screening test, and HbA1c (p > 0.05).

**Table 2:** Multiple linear regression analysis including nesfatin-1, gestational age, BMI, age, fasting plasma glucose, and HOMA-IR

	Standardized coefficient (β)	p value
Gestational age	0.021	0.553
Age	0.014	0.197
BMI	0.032	0.048^a^
Nesfatin-1	-0.026	0.014^a^
HOMA-IR	-0.038	0.423
Fasting plasma glucose	0.008	0.054

β coefficients and p values are given.

Dependent variable; gestational diabetes.

Independent variables; nesfatin-1, gestational age, BMI (body mass index), age, fasting plasma glucose, and HOMA-IR (homeostasis model assessment insulin resistance index).

^a^ Statistically significant (p < 0.05).

**Table 3:** The correlation analyses of nesfatin-1 with some parameters in women with gestational diabetes

	p value	r
Age	0.19	0.09
BMI	0.46	0.12
Fasting insulin	0.14	0.25
Fasting blood glucose	0.80	-0.04
HOMA-IR	0.22	-0.21
1-h glucose (50 g OGTT)	0.18	-0.23
HbA1c	0.308	-0.13
Gestational age	0.015^a^	0.30

BMI: body mass index; HOMA-IR: homeostasis model assessment index.

^a^ The correlation between nesfatin 1 and gestational age was statistically significant in gestational diabetes (p < 0.05).

**Figure 1:**
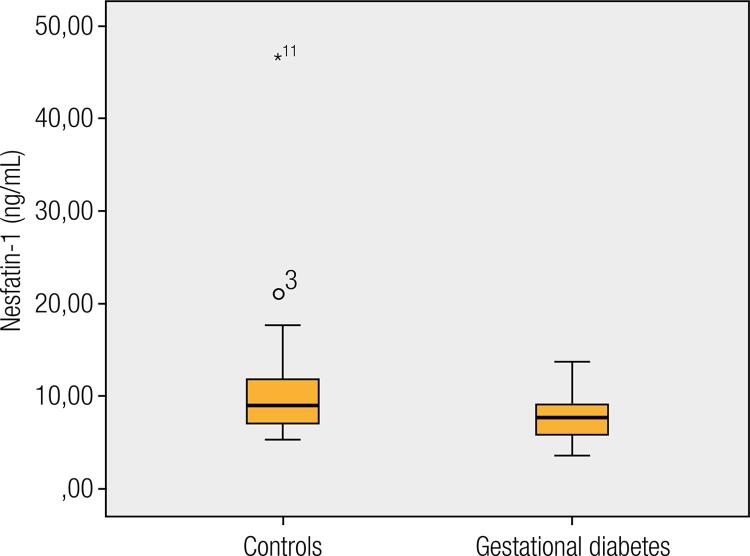
Distribution of serum nesfatin-1 concentration in controls and in gestational diabetes. 97 x 54 mm (300 x 300 DPI).

## DISCUSSION

In the present study, we demonstrated that serum nesfatin-1 levels at 24-28 weeks of gestation are significantly lower in women newly diagnosed with GDM compared to healthy pregnant women. In 2010, Aydın S. first described the presence of nesfatin-1 in breast milk (10). In that study, nesfatin-1 was investigated in serum, milk, and colostrum of lactating women who were diagnosed with GDM and in healthy lactating women. Nesfatin-1 was demonstrated to be lower in the serum of lactating women diagnosed with GDM than that of the control participants. It was suggested that the breast tissue was likely to be a source of nesfatin-1 just like the central nervous system, adipocytes, gastric endocrine cells, and pancreatic beta cells. It was also pointed out that this may thus be important for growth, energy regulation, and maturation of the gastrointestinal system in neonates.

Clinical significance of nesfatin-1 in various metabolic diseases like obesity, Type 2 DM, and insulin resistance was demonstrated in several studies in the literature, but its effects on GDM are unknown. The literature reports only one study about the nesfatin-1 levels in gestational diabetes. In this study, Aslan and cols. assessed both serum and cord blood apelin and nesfatin-1 levels in pregnant women with GDM and in healthy pregnant women (11). Cord blood nesfatin-1 and apelin-36 levels were comparable between women with GDM and controls, serum apelin-36 concentrations were found to be higher, and serum nesfatin-1 concentrations were found to be lower in women with GDM compared to controls. The designs differ between our study and that study in some aspects, including the time of nesfatin-1 sampling and patient selection. In the study of Aslan and cols., the study group was composed of pregnant women who were being followed with diagnosis of GDM and whose blood glucose levels were strictly controlled, whereas our study included pregnant women who were newly diagnosed with GDM so that none of them were on restricted diets or using insulin therapy. Additionally, Aslan and cols. did not point out whether the women diagnosed with GDM were using an insulin regimen or only following a specific diet. They measured nesfatin-1 levels at the time of birth before placenta delivery in patients with GDM and in controls. Differently, we measured nesfatin-1 at 24-28 weeks of pregnancy in control and study groups. In 2012, Boutsikou and cols. measured cord blood nesfatin-1 and insulin concentrations in 40 large (9 born from diabetic and 31 from non-diabetic mothers) and 20 appropriate for gestational age pregnancies (12). Cord blood nesfatin-1 concentrations were shown to be significantly lower in large gestational age pregnancies compared to appropriate for gestational age pregnancies. Furthermore, fetal nesfatin-1 concentrations were found to be elevated in infants born from mothers with GDM compared to those born from mothers without GDM.

Serum nesfatin-1 levels in obesity and Type 2 DM reported in the literature was rather controversial. In 2010, Tsuchiya and cols. reported that fasting concentrations of nesfatin-1 were significantly lower in persons with high BMI compared to non-obese participants (13). However, in contrast to this study, Ramanjaneya and cols. found increased nesfatin-1 levels in obese states in both rodents and humans in their study (14). In a recent study, Gonzalez and cols. demonstrated that nesfatin-1/NUCB2 mRNA expression in the pancreatic islets is markedly increased in Goto-Kakizaki rats, a model with Type 2 DM, characterized by impaired insulin secretion and visceral fat accumulation (15).

Involvement of nesfatin-1 in the regulation of insulin secretion remains largely unknown. In 2013, Nakata and cols. reported that nesfatin-1 exerts its metabolic effects partly via promoting insulin release by Ca^+2^ influx through L-Type Ca^+2^ channels independently of protein kinase A (PKA) and phospholipase A_2_ (PLA_2_) so that dysregulation of nesfatin-1 might be implicated in metabolic disorders, particularly in Type 2 DM (16). In 2010, Su and cols. reported that intravenous injection of nesfatin-1 suppresses hyperglycemia in ob/ob mice by enhancing insulin secretion (17). In another study, Li and cols. demonstrated that fasting nesfatin-1 levels were found to be decreased in persons with Type 2 DM compared to healthy participants and persons with Type 1 DM (8). Additionally, it was pointed out that although this result is unclear, nesfatin-1 may be a causal factor in diabetic hyperphagia. In 2012, in contrast to the study of Li and cols., Zhang and cols. demonstrated elevated levels of nesfatin-1 in participants with Type 2 DM and with impaired glucose tolerance (IGT) compared to controls (18). It was shown that plasma nesfatin-1 was positively correlated with BMI, HbA1c, fasting blood glucose, fasting plasma insulin, and HOMA-IR. In a recent study, Ding and cols. revealed that Type 2 DM patients with peripheral arterial disease (PAD) exhibited marked lower serum nesfatin-1 concentrations than those without PAD. They hypothesized that serum nesfatin-1 levels were inversely correlated with the development and severity of PAD in Type 2 DM patients (19).

Previous studies reveal conflicting data about the correlations of nesfatin-1 with metabolic parameters. For instance, Zhang and cols. showed that nesfatin-1 was positively correlated with BMI, HbA1c, fasting blood glucose, fasting insulin, and HOMA-IR in participants with newly diagnosed Type 2 DM (18). In contrast to that study, Deniz and cols. revealed a negative correlation between nesfatin-1 and BMI, fasting blood glucose, and HOMA-IR in patients with polycystic ovary syndrome, an endocrine disorder commonly presenting with obesity and insulin resistance as well as hyperandrogenemia and hirsutism (20). Ding and cols. also revealed a negative correlation between nesfatin-1 and BMI in their study (19). In the present study, we did not find any correlation between nesfatin-1 and fasting glucose, 1-h glucose after 50-g glucose screening test, 1-h, 2-h, and 3-h glucose after 100-g oral glucose tolerance test, HbA1c, HOMA-IR, and BMI. We found a significant positive correlation between nesfatin-1 and gestational age, contrary to the results of the study by Aslan and cols., as they had observed a negative correlation between nesfatin-1 and gestational age (11).

Because few data are currently present in the literature, the circulating levels of nesfatin-1 and its correlation with several parameters in GDM are not well-known and might require further investigation. Measurement of serum nesfatin-1 consecutively during the gestation may provide more evidence about the role of nesfatin-1 in GDM in future research.

In conclusion, we have demonstrated for the first time reduced nesfatin-1 levels at 24-28 weeks of pregnancy in women newly diagnosed with gestational diabetes. Although the significance of this result is yet unclear, it is important because it draws attention to nesfatin-1 levels in GDM and may shed light on further research in this area.
